# Behavioral measures of attention and cognitive control during a new auditory working memory paradigm

**DOI:** 10.3758/s13428-019-01308-z

**Published:** 2019-12-03

**Authors:** Jürgen Kayser, Lidia Y. X. Wong, Elizabeth Sacchi, Lindsey Casal-Roscum, Jorge E. Alvarenga, Kenneth Hugdahl, Gerard E. Bruder, John Jonides

**Affiliations:** 1 Division of Cognitive Neuroscience, New York State Psychiatric Institute, New York, NY USA; 2 Department of Psychiatry, Columbia University Vagelos College of Physicians & Surgeons, New York, NY USA; 3 Division of Translational Epidemiology, New York State Psychiatric Institute, New York, NY USA; 4 Department of Biological and Medical Psychology, University of Bergen, Bergen, Norway; 5 Division of Psychiatry, Haukeland University Hospital, Bergen, Norway; 6 Department of Radiology, Haukeland University Hospital, Bergen, Norway; 7 Department of Psychology, University of Michigan, Ann Arbor, MI USA

**Keywords:** Attention, Auditory modality, Proactive control, Test–retest reliability, Working memory

## Abstract

Proactive control is the ability to manipulate and maintain goal-relevant information within working memory (WM), allowing individuals to selectively attend to important information while inhibiting irrelevant distractions. Deficits in proactive control may cause multiple cognitive impairments seen in schizophrenia. However, studies of cognitive control have largely relied on visual tasks, even though the functional deficits in schizophrenia are more frequent and severe in the auditory domain (i.e., hallucinations). Hence, we developed an auditory analogue of a visual ignore/suppress paradigm. Healthy adults (*N* = 40) listened to a series of four letters (600-ms stimulus onset asynchrony) presented alternately to each ear, followed by a 3.2-s maintenance interval and a probe. Participants were directed either to selectively ignore (I) the to-be-presented letters at one ear, to suppress (S) letters already presented to one ear, or to remember (R) all presented letters. The critical cue was provided either before (I) or after (S) the encoding series, or simultaneously with the probe (R). The probes were encoding items presented to either the attended/not suppressed ear (“valid”) or the ignored/suppressed ear (“lure”), or were not presented (“control”). Replicating prior findings during visual ignore/suppress tasks, response sensitivity and latency revealed poorer performance for lure than for control trials, particularly during the suppress condition. Shorter suppress than remember latencies suggested a behavioral advantage when discarding encoded items from WM. The paradigm-related internal consistencies and 1-week test–retest reliabilities (*n* = 38) were good to excellent. Our findings validate these auditory WM tasks as a reliable manipulation of proactive control and set the stage for studies with schizophrenia patients who experience auditory hallucinations.

**Electronic supplementary material:**

The online version of this article (10.3758/s13428-019-01308-z) contains supplementary material, which is available to authorized users.

The ability to selectively attend to goal-relevant information is essential to a properly functioning working memory (WM) system (see, e.g., see Jonides & Nee, 2006, for a review). This skill, referred to as proactive control, allows individuals to disregard task-irrelevant information while simultaneously maintaining and updating information needed to achieve specific goals. Proactive control has been empirically connected to performance on various WM tasks, such as those related to WM span (e.g., reading span task; Whitney, Arnett, Driver, & Budd, 2001). In the realm of neuropsychiatric disorders, proactive control has been identified as a putative source of multiple cognitive impairments seen in patients diagnosed with schizophrenia (Barch & Ceaser, 2012). Indeed, impairments in both cognitive control and WM are among the most frequently cited cognitive deficits associated with schizophrenia (e.g., Barch, 2005; Barch et al., 2004; Smith, Eich, Cebenoyan, & Malapani, 2011).

In the context of WM, cognitive control has been successfully examined in both healthy and psychiatric populations using a combination of tasks that require participants to either (1) prevent (ignore) irrelevant information from entering WM or (2) retroactively suppress irrelevant information that has already entered WM (Nee & Jonides, 2008, 2009; Smith et al., 2011). These so-called ignore/suppress tasks have been successfully used with schizophrenia patients (Eich, Nee, Insel, Malapani, & Smith, 2014; Smith et al., 2011) and have also been identified by the Cognitive Neuroscience Treatment Research to Improve Cognition in Schizophrenia (CNTRICS) consortium as one of two promising WM paradigms to study interference control (goal maintenance being the other) for the development of neuroimaging biomarkers in schizophrenia (Barch, Moore, Nee, Manoach, & Luck, 2012).

The purpose of the ignore/suppress tasks is to distinguish between perceptual encoding and maintenance of information within WM. In each task, participants are presented with a series of items (the encoding set), which is then followed by a brief delay (maintenance interval). The maintenance interval is followed by a probe, which consists of a stimulus that either was or was not present during encoding. The participant’s task is to indicate whether the probe was included within a targeted subset of the encoding set. The critical difference between the ignore and suppress tasks lies in *when* (relative to encoding) the participant is given instructions on how to process (i.e., either ignore or suppress) items from the encoding set. In a typical example of this task, participants are presented with a list of words displayed in one of two different colors (e.g., teal or blue; Nee & Jonides, 2009). In the ignore task, *prior* to the start of encoding, participants are instructed to ignore any stimulus presented in one of the two colors (e.g., ignore all words presented in teal). In contrast, instructions for the suppress task do not appear until *after* all items from the set have been encoded and stored in WM (e.g., suppress information about items that were presented in teal when responding to the probe). The timing of the instructions allows for the dissociation of bottom-up attentional or perceptual processing of encoding items (via ignore) and the top-down cognitive/proactive control processes (via suppress). That is, the ignore task taps into the ability to filter out items that are task-irrelevant, whereas the suppress task measures the ability to actively manipulate information already held within WM.

The measurement of how well participants can either filter irrelevant information (ignore) or manipulate information stored in WM (suppress) lies in responses to three separate types of probes (see also Fig. 1 below). Probes that were presented during encoding and selectively attended are considered “valid.” The critical comparison is between probes that were not presented at all during encoding (“controls”) and probes that were presented during encoding but were not selectively attended (“lures”). Responses to both lure and control items are considered *negative*—when presented with either of these items, the correct response is *no*, the item was *not* part of the encoding set to which the participant should have been attending. The extent to which responses to lures and controls differ offers insight into how well the participant effectively (1) inhibited irrelevant information from entering WM or (2) manipulated information already stored in WM.

**Fig. 1:**
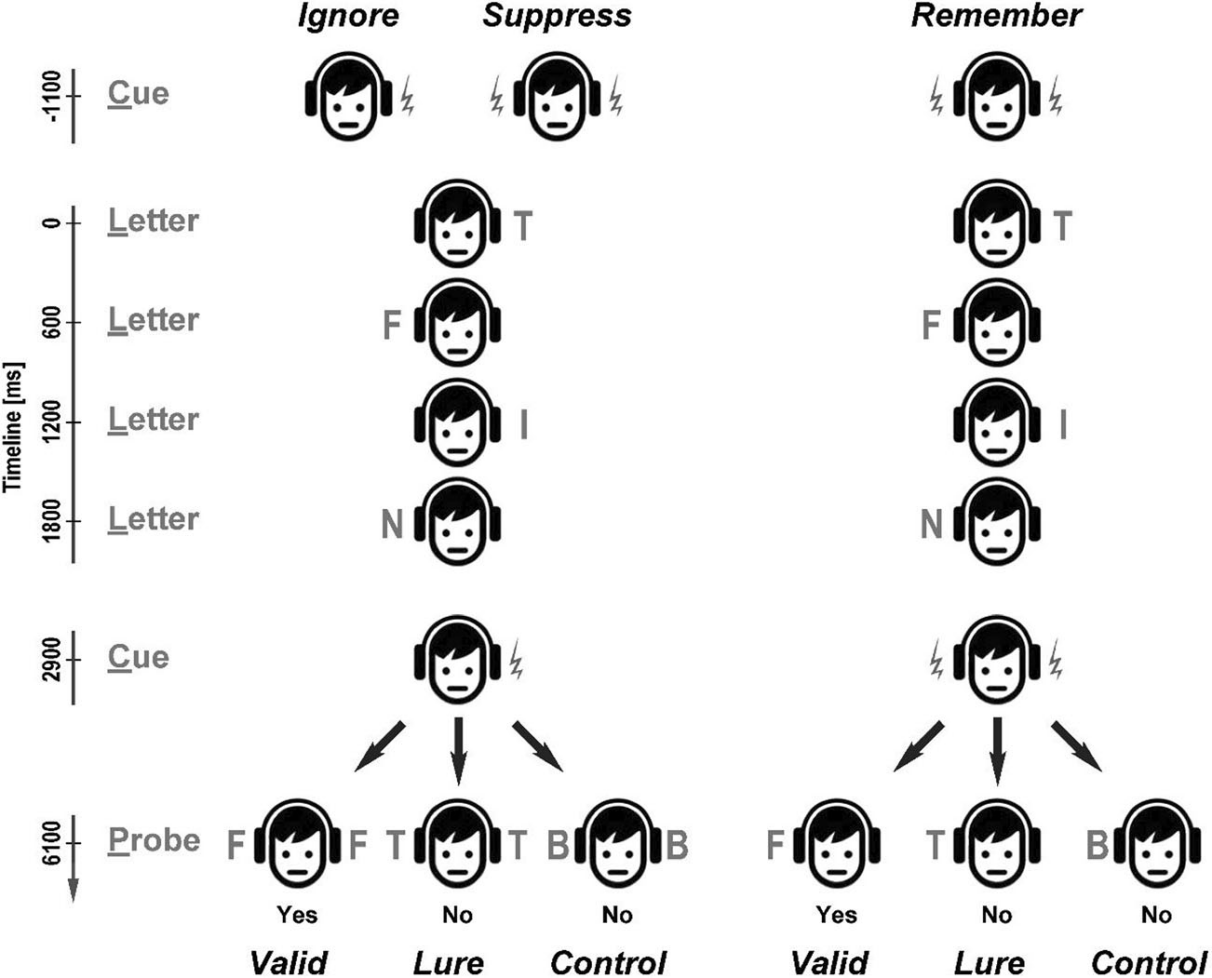
Schematic for the ignore, suppress, and remember auditory working memory tasks. Letters indicate monaural encoding and binaural probe stimuli (next to the ear presented to). Monaural and binaural cue stimuli (buzz sounds) are indicated by a flash icon.

Behavioral results from these tasks in healthy populations reveal longer reaction times (RTs) and increased error rates in response to lure as compared to control trials. Furthermore, this difference is more pronounced in the suppress task than in the ignore task (e.g., Monsell, 1978; Nee & Jonides, 2008; Smith & Jonides, 1998). Smith et al. (2011) used the ignore/suppress paradigm to investigate perceptual and memorial selection processes in schizophrenia. In this study, 24 healthy controls (HC) showed poorer performance during suppress than ignore, as reflected by increased RTs and error rates for lure over control items. As compared to HC, 17 schizophrenia patients (SZ) showed a similar response pattern, and critically had particularly poorer performance in the suppress than ignore task, which Smith et al. interpreted as a deficit in selecting or manipulating information within WM. Eich et al. (2014) substantially replicated the behavioral suppress effects for 18 SZ patients.

Although most studies using visual ignore/suppress tasks have focused on verbal WM, comparable effects can also be observed with visual objects (Cyr et al., 2017). In sum, visual ignore/suppress tasks have proven to be powerful tools for studying the distinct roles of top-down cognitive control and bottom-up perceptual selection in the context of WM processing. For these reasons, these tasks have been identified as a particularly useful paradigm for the study of inhibitory control in patients with schizophrenia, assisting ongoing efforts to identify behavioral and neuroimaging markers that are predictive of treatment outcomes (Barch et al., 2012).

However, these tasks have not been employed in any context other than the visual modality, and yet, the examination of proactive control in the auditory domain would be of *particular* interest for studying cognitive impairments in schizophrenia, as patients show greater deficits in the auditory as opposed to the visual modality (e.g., Pelletier, Achim, Montoya, Lal, & Lepage, 2005). Electrophysiologic abnormalities in schizophrenia, particularly reductions of the P3 component thought to reflect cognitive processing, are more robust and common with tasks using auditory than visual stimuli (e.g., Ford, 1999; Jeon & Polich, 2003; Kayser, Tenke, Gil, & Bruder, 2009). These deficits may be directly related to the close association between language and phonological representations (Crow, 2004), the phonological code itself closely tied to working memory processes (Baddeley, 1983), and possibly to a higher occurrence of auditory than visual hallucinations in schizophrenia (Ford, 1999). Indeed, it has been proposed that auditory hallucinations in schizophrenia may result from a deficit in cognitive control (e.g., Badcock & Hugdahl, 2014; Hugdahl, 2009; Lewis-Hanna, Hunter, Farrow, Wilkinson, & Woodruff, 2011). An auditory ignore/suppress paradigm could therefore be of value for understanding the cognitive mechanisms behind auditory hallucinations.

Further motivation for the examination of auditory processing stems from notable differences in information processing that exist between auditory and visual modalities. In contrast to the visual system, where information is immediately available at the onset of stimulus presentation, auditory stimulus analysis requires the rapid and highly sequential temporal integration of sound (e.g., Ungerleider, Courtney, & Haxby, 1998; Ungerleider & Haxby, 1994). The implementation of a task that takes advantage of the sequential nature of auditory processing could aid in revealing mechanisms underlying specific deficits in patients with schizophrenia, involving receptive language or phonological processing, which have been hypothesized to be at the core of the disorder (e.g., Angrilli et al., 2009; Condray, 2005; Crow, 1997).

In the present study, we aimed to develop a feasible paradigm to examine cognitive control in the context of WM within the auditory domain. The auditory WM tasks were optimized for simultaneous recordings of event-related potentials (ERP) and oscillations (ERO), as these measures would likely reveal critical information on the role of “bottom-up” sensory or perceptual processes and “top-down” attentional or cognitive control processes in WM and their underlying brain mechanisms.^1^ The implemented tasks combine aspects of a serial position test (SPT) and dichotic listening tasks, which have revealed behavioral (Bruder et al., 2011) and ERP abnormalities in schizophrenia (Bruder et al., 1999; Kayser et al., 2006), along with the central aspect of visual ignore/suppress tasks, which engage cognitive control during WM processing.

In addition to examining auditory WM during ignore/suppress tasks, we aimed to further previous work through the inclusion of a novel task. Motivation for this stems from concerns regarding the interpretation of impaired performance on the suppress task relative to the ignore task, which applies to both healthy individuals and patients with schizophrenia (e.g., Nee & Jonides, 2008, 2009; Smith et al., 2011). In both the original visual tasks (and the proposed auditory tasks), the suppress task requires participants to maintain information about *both* the items from the encoding set (i.e., the content) *and* information regarding the context in which the item is to be remembered (e.g., which item was presented *and* in which color or location). This is also known as content–context binding (e.g., Cyr et al., 2017; Nee & Jonides, 2013). Contextual (or source) memory is not called on in the same way during the ignore task, in which contextual information can be disregarded prior to encoding, maintenance or retrieval. The additional memory load required to maintain and recall contextual information could result in poorer performance on the suppress task and thereby constitutes a potential confound.

Hence, we opted to include an auditory implementation of the original item-recognition task (Sternberg, 1966, 1969), given that the ignore and suppress tasks are variants of this item-recognition memory task (Smith et al., 2011). In this “remember” task, participants were instructed to remember *all* items in the encoding set *prior* to the start of encoding. This additional task was intended to serve as a control for the role of WM load as well as contextual (source) memory during the suppress task. The extent to which performance differs between suppress and remember tasks will demonstrate whether discarding irrelevant items already encoded in WM provides a distinct advantage during retrieval, as encoding demands are identical for suppress and remember tasks.

The overarching goal of the present study was to introduce a feasible test of proactive control during a WM paradigm within the auditory domain. Accordingly, we report behavioral evidence from new auditory WM tasks designed to emulate visual ignore/suppress tasks (e.g., Nee & Jonides, 2008, 2009). We sought (1) to determine whether the same pattern of behavioral results found in prior work with healthy individuals using visual tasks extends into the auditory domain, and (2) to assess the psychometric properties of the auditory tasks (i.e., internal consistency and temporal stability of behavioral performance measures) by administering comparable versions of these tasks to the same participants after one week. At the same time, we also expanded this paradigm by including an auditory analogue of an item-recognition (remember) task as a specific control condition for the suppress task. Achieving these goals would establish this new paradigm as a useful tool for examining and distinguishing “bottom-up” and “top-down” cognitive control processes in auditory WM, which would be of considerable value for future research with both healthy and psychiatric populations.

Specifically, we predicted poorer performance (decreased response accuracy, increased response latencies) during the suppress task than during the ignore task, and for lure than for control items. We further hypothesized that this condition difference would be greater during the suppress task than during the ignore task, as has been previously reported for the visual implementation of these tasks (e.g., Monsell, 1978; Nee & Jonides, 2008; Smith et al., 2011; Smith & Jonides, 1998). It was expected that these critical effects are robust and will therefore yield strong test–retest reliability and high internal consistency. With regard to the novel remember task, we hypothesized poorer performance for the remember as compared to the suppress task due to increased WM load at item retrieval.

## Materials and methods

### Participants

The sample^2^ consisted of 40 healthy, right-handed adults (Table 1) between 18 and 55 years of age, who had been recruited from the New York metropolitan area through online advertisements on Craigslist and via the Psychophysiology Laboratory website at New York State Psychiatric Institute (NYSPI). Two participants (one female, one male) failed to return for the 1-week retest session. Although scheduling allowed for a range between 6 and 12 days after the initial test session, most participants (*n* = 27) were retested after 7 days (*M* ± *SD*, 7.4 ± 1.2), and all at about the same time of the day (*M* ± *SD*, Session 1 vs. 2, 12:48 h ± 1:53 h vs. 12:56 h ± 2:08 h; time difference, 0:08 h ± 1:00 h).

**Table 1:** Summary of demographic variables

		Healthy Adults (*N* = 40)		
		Mean/*n*	*SD*	Range
Sex				
Female		21		
Male		19		
Age (years)		32.0	10.9	18–55
Race/Ethnicity				
Native American		1		
Asian		8		
Black/African American		12		
White/Caucasian		14		
More than one race		5		
EHI LQ ^a^		78.17	23.80	20–100
Education (years)		15.4	1.95	12–19
NART ^b^		35.4	9.29	16–55
Parental SES ^c^		45.15	16.62	7.5–66

^a^Edinburgh Handedness Inventory (Oldfield, 1971) laterality quotient, which can vary between −100.0 (*completely left-handed*) and +100.0 (*completely right handed*). ^b^ National Adult Reading Test (Bright, Jaldow, & Kopelman, 2002). ^c^ Parental socioeconomic status (Hollingshead, 1975), *n* = 36.

All participants were required to speak English well enough to comprehend and to comply with protocol requirements, as determined by phone screening. This was followed-up by a Structured Clinical Interview for DSM-IV Axis I Disorders (First, Spitzer, Gibbon, & Williams, 1996) administered by trained personnel to exclude participants with Axis I disorders or any other psychopathology, if they met DSM-IV criteria for substance abuse or dependence (including alcohol) in the last 6 months, or for any of the following reasons: seizures, hearing loss exceeding 30 dB (standard audiogram) or difference between ears being larger than 15 dB, history of significant head trauma or other neurological disorders, or lack of capacity to give informed consent. Risks and benefits were fully explained to all eligible individuals, and written informed consent was obtained from all participants. Out of 92 volunteers who passed the phone screening, 17 did not show for the scheduled clinical interview and 27 failed to pass. From the 48 volunteers who passed the clinical interview, one was excluded because of hearing loss, one was unable to comprehend the task instructions, and six opted not to continue with the electroencephalogram (EEG) recordings. Participants were instructed to arrive for their EEG sessions well-rested, and, at the day of testing, to avoid extreme mental and physical activity and to refrain from using alcohol, caffeine and nicotine; compliance was verified via administration of a baseline questionnaire at the beginning of each session. The research protocol was approved by the NYSPI Institutional Review Board.

### Procedure

A schematic comparison of the timeline for the three different auditory WM tasks is shown in Fig. 1. Each trial began with the presentation of a gray speaker icon over a lighter gray background to reduce eye movements and to focus attention. This icon was centered on a 20-in. LED monitor (approximate eye distance 1 m) and remained visible until the end of the trial (i.e., after the response). Participants were instructed to look at the icon throughout the trial while trying to minimize eye and body movements.

After 500 ms, an auditory cue was presented, consisting of a 350-ms buzz sound (i.e., a 38-Hz square wave complex tone with a linearly tapered 20% rise and fall time; see flash icon in Fig. 1). For the ignore task, this cue was presented to the left *or* right ear (monaural buzz) to indicate to the participant which ear to ignore; in contrast, for the suppress and remember tasks, the cue was presented to *both* ears (binaural buzz). After an 1,100-ms delay (i.e., after cue onset), a series of four spoken letters was presented, with letters presented in alternating order to the left or right ear using a 600-ms stimulus onset asynchrony (SOA); the initial ear was alternated across trials in a pseudo-random order. After another 1,100 ms (i.e., after onset of the last letter in the series), a second auditory cue was presented to the left *or* right ear (monaural buzz) for the ignore and suppress tasks, whereas a binaural buzz was presented in the remember task. For ignore, this cue was a mere repetition of the first cue; for suppress, this cue indicated which ear to suppress (i.e., which items were not relevant for the correct response).^3^

**Fig. 2:**
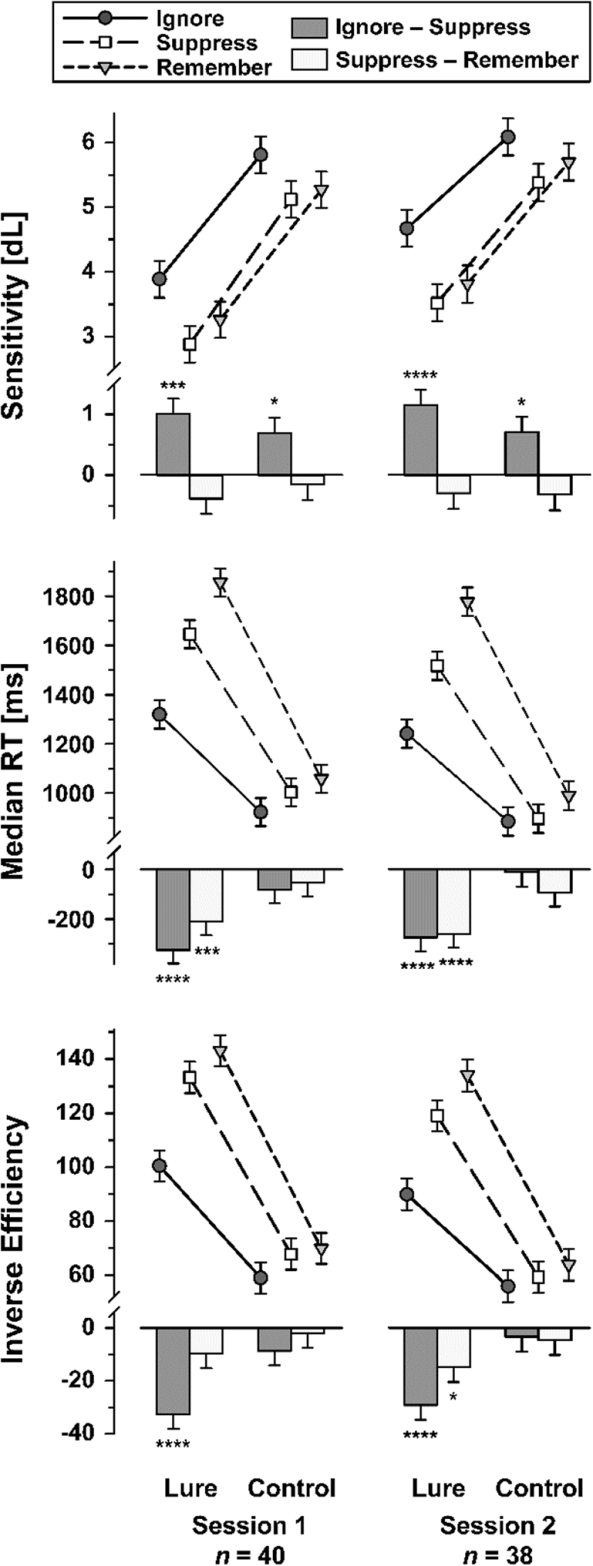
Mean (± *SEM*s) behavioral performance measures, reflecting response sensitivity [d_L_] (A), median response latency [ms] (B), and inverse efficiency [ms/(d_L_ + 10)] (C) for each condition (control, lure) and task (ignore, suppress, remember), plotted separately for each session. Graphs depict the estimated means using an omnibus model including all three tasks (see the text). The condition means (line graphs) are supplemented by planned pairwise differences (bar graphs) showing ignore-minus-suppress and suppress-minus-remember, with significant effects (Tukey-adjusted for a family of three tests) marked as ^*^p ≤ .05, ^***^p ≤ .001, ^****^p ≤ .0001.

After a 3,200-ms delay interval, a binaural probe letter was presented for ignore and suppress tasks. For the remember task, a monaural probe was presented that indicated the to-be-remembered ear (i.e., the set of valid letters). Participants were instructed to press a “Yes” or “No” button to indicate whether the probe letter matched a letter in the memory set (i.e., the to-be-attended [not ignored] or remembered [not suppressed] ear). There were three probe conditions: (1) the probe could match one of the letters that should still be in WM (40% of the trials, valid; the letter “F” in Fig. 1); (2) it could match one of the letters that should have been ignored or suppressed (30%, lure; “T”); or (3) it did not match any of the letters presented on that trial (30%, control; “B”). After 2,500 s (i.e., after probe onset), a blank screen (1,000-ms duration) signaled the end of this trial, providing approximately a 3-s response window after probe presentation.

The comparison of accuracy and latencies for negative responses to lure and control trials (i.e., correct answer is “No”) provided an index of cognitive control. If selection of only letters on the ear to be attended or remembered were perfect, there should be no difference between performance for lures and controls. To the extent that selection is poor, the difference between these two types of negative responses should be large.

The stimuli were generated with a text-to-speech software engine (AT&T Labs, Inc.–Research, 2011) and software (Morozov, 2013). For each trial, different letters were randomly selected from 15 monosyllabic stimuli (A to F, I to O, R to U) using only clearly perceptible speech sounds (mean duration 387 ± 42 ms, range = 311–464 ms; see Supplementary Fig. S1). However, each four-letter encoding sequence was limited by the following constraints. Consecutive letters could not (1) begin or end with the same vowel (e.g., [be:]–[de:]), (2) be alphabetically consecutive (e.g., A-B), or (3) repeat within three consecutive trials.

All stimuli were presented via headphones at about 70 dB SPL using Presentation software version 18.2 (Neurobehavioral Systems, Inc., 2007). Trials were arranged in nine 30-trial blocks (three ignore [I], three suppress [S], three remember [R], with task order systematically counterbalanced across participants using Latin squares of rank 3 [e.g., SRI–RIS–ISR]). Blocks were separated by short rest periods (about 1–2 min), during which participants were reinstructed for the upcoming task. In each block, the first item was a left or right ear presentation (pseudo-random), with half of the trials beginning with either ear. All conditions (40% valid, 30% lure, 30% control) and ear (left, right) were pseudo-randomly distributed for each task. Participants responded by pressing one of two designated buttons on a Cedrus RB-830 response pad using their preferred hand (all were right-handed). Participants were familiarized with each task after completion of their clinical interview and at the beginning of each session via short training blocks (three trials), and only continued after demonstrating adequate comprehension of all task instructions (i.e., correctly responding after the probe in at least two out of three training trials).^4^

During the retest session, each participant received a different permutation (i.e., task order and trial sequence). However, these alterations were not random but systematically counterbalanced across participants. Using the permutation for Sequence 1, Sequences 2 and 3 were realized by shuffling the tasks (ignore [I] becomes suppress [S] and then remember [R], S becomes R and then I, and R becomes I and then S, respectively). Sequences 1 to 3 were repeated as Sequences 4 to 6, however, the side of monaural stimulation was reversed (left ear becomes right ear and vice versa) together with also reversing the to-be-attended ear. This systematic was repeated with a different permutation for Sequences 7 to 12 and so forth. Test–retest sessions were realized by combining sequence numbers differing by 6 (e.g., 1 and 7, 8 and 2, etc.; Supplement A).

Of note, the only difference among the three tasks was when the critical instruction was given relative to the memory set, that is, either before (ignore) or after (suppress) the encoding series, or with the probe (remember), allowing for a systematic manipulation of auditory WM processes. However, the stimulus sequence was identical from the first encoding letter to the probe letter. Ignore and suppress tasks differed only in the first auditory cue, whereas suppress and remember tasks differed only in the second auditory cue up to the probe; however, the monaural probe was unique to the remember task. Although the ignore and suppress tasks are close analogues to visual ignore/suppress tasks, the additional remember task adds an auditory item-recognition equivalent as a control condition to the suppress task. This task does not require participants to suppress items in one ear during the maintenance phase (i.e., inhibit information already stored in WM), but just to retrieve the letters presented in the cued ear (i.e., source memory information is critical). This third control task, which was specifically designed for the concurrent EEG recordings (i.e., sustained event-related potentials and oscillations), should help to both address the role of source memory and reveal differences in brain activation between suppress and remember during the maintenance phase (i.e., after the second cue).

### Data transformation and analysis

For the analysis of behavioral data, response latency was characterized as the individual median reaction time (RT_med_) for correct responses only. To assess accuracy, a *d'*-like measure of sensitivity (*d*_L_) was calculated using hit rates and false alarms (see Snodgrass & Corwin, 1988, pp. 35–37), which eliminates response bias confounds. “Yes” and “no” responses to valid items were considered hits and misses, respectively, whereas responses to lure and control items provided two types of false alarms and correct rejections, allowing for the computation of separate false alarm rates for lure and control trials (Table 2; Supplement B).

**Table 2:** Signal detection theory diagram for the auditory working memory paradigm

			Response		
			*Yes*	*No*	
Probe	Valid	*Yes*	Hit	Miss	Hit Rate
	Lure	*No*	FA	CR	FA Rate _lure_
	Control	*No*	FA	CR	FA Rate _control_

FA: false alarm; CR: correct rejection.

To account for trade-offs between speed and accuracy, an inverse efficiency score was also calculated for each subject, task, condition, and session (e.g., Bruyer & Brysbaert, 2011; Townsend & Ashby, 1978, 1983). Inverse efficiency scores were calculated as the ratio of RT_med_ to *d*_L_ + 10, effectively scaling response latency by sensitivity and thereby allowing for their simultaneous analysis (i.e., higher inverse efficiency scores indicate less efficient performance; Supplement B).^5^

Several considerations motivated the decision to analyze and report aggregated rather than individual response latencies (RTs). First, because the sensitivity measure cannot be calculated for individual responses, findings for accuracy and inverse efficiency will not be directly comparable to these of RTs. Second, multilevel modeling (MLM) of individual RTs will not allow a comparison of the present behavioral findings with those of previous reports, all of which used aggregated performance measures. Given that the main purpose of this report was to establish the validity and usefulness of a new paradigm, using a different analytic approach would be counterproductive. Third, a median-based normalization is not possible when using individual RTs, although other normalization transformations (e.g., log transform) could be applied. Lastly, after performing additional MLM analyses for individual RTs, the overall findings were highly comparable to those stemming from the aggregated data.

### Interclass correlation coefficients

To determine the appropriateness of a mixed-model analysis for the current dataset, *unconditional* interclass correlations (ICCs; Bickel, 2007; McGraw & Wong, 1996) were computed for each of the three dependent variables for each task from the intercept and residual variance obtained for a one-way baseline model with subject as a random factor (i.e., intercept variance divided by the sum of the residual and intercept variance) to obtain *rho* (*ρ*) (see Mathieu, Aguinis, Culpepper, & Chen, 2012; Supplement B)*.* For the majority of the planned models, ICCs were greater than .1 (.074 ≤ *ρ* ≤ .519; see Supplementary Table T1 for further details, including *t* statistics, and Supplement B for the R code), suggesting that measurements in response to each task/condition combination per subject were not independent. Accordingly, analyses were performed using multilevel regression modeling.

### Analysis approach and model specifications

The critical comparison between ignore and suppress tasks was examined using a model that included fixed factors of *task* (ignore, suppress), *condition* (lure, control), and *session* (1, 2). Task, condition, and session were allowed to interact. Subject was entered as a random factor (random intercepts only).^6^ To assess the impact of task and condition in each session separately, as future studies will likely be based on a single test session (e.g., when including psychiatric populations), analogous follow-up models were run on data from each session (i.e., dropping session as a design factor). To resolve the source of significant interactions, follow-up analyses involved simple effects and pairwise comparisons. The same analysis approach was used for the comparison of the suppress and remember tasks. Effect sizes were estimated using semipartial *R*^2^ for individual effects (see Edwards, Muller, Wolfinger, Qaqish, & Schabenberger, 2008).

To visualize the findings (i.e., results from mixed models using subject as a random factor), all figures depict adjusted (i.e., subtracting the random intercept for each subject) rather than raw data. Random intercepts used to make these figures were extracted from an omnibus model including all three tasks (ignore, suppress, remember), each condition of interest (lure, control), and both sessions (see Supplementary Table T2 for a summary of these mixed-model effects). This allowed viewing the data in a way that is consistent with the current analytic approach.

All statistical analyses described above were performed in R (version 3.6.0; R Core Team, 2015), using the linear mixed-effect regression (LMER) framework as implemented via the lme4 package (version 1.1-21; Bates, Kliegl, Vasishth, & Baayen, 2015a; Bates, Mächler, Bolker, & Walker, 2015b). Since the data and models analyzed did not contain fixed between-subjects effects, restricted maximum likelihood (REML) estimation was used (see, e.g., Haverkamp & Beauducel, 2017). All effect means, corresponding standard errors, simple effects and pairwise comparisons were estimated within the LMER framework via the emmeans package (version 1.3.4; Lenth, Singmann, Love, Buerkner, & Herve, 2019; Supplement B).

Test–retest reliability (Spearman–Brown-corrected Pearson correlations)^7^ and internal consistency (Cronbach’s *α*) were calculated with SPSS (version 23.0; SPSS Inc., 2015).

## Results

### Ignore versus suppress

Table 3 provides a summary of mixed-model effects, comparing the ignore and suppress tasks across both sessions and separately for each session.

**Table 3:** Summary of linear mixed-effect regression models comparing ignore and suppress tasks

		Sensitivity (*d*_L_)					Median Response Latency					Inverse Efficiency				
	*df*_1_	*df*_2_	*F*	*p*		*R*^2^_β_	*df*_2_	*F*	*p*		*R*^2^_β_	*df*_2_	*F*	*p*		*R*^2^_β_
Sessions 1 & 2																
T	1	265.1	48.3	<.0001	****	.154	265.0	40.6	<.0001	****	.133	265.1	43.4	<.0001	****	.141
C	1	265.1	212.2	<.0001	****	.444	265.0	344.0	<.0001	****	.565	265.1	320.0	<.0001	****	.547
S	1	266.8	14.0	.0002	***	.050	266.9	10.8	.001	**	.039	267.1	10.3	.001	**	.037
T×C	1						265.0	21.8	<.0001	****	.076	265.1	19.7	<.0001	****	.069
T×S																
C×S	1	265.1	2.95	.09	(*)	.011										
T×C×S																
Session 1																
T	1	117	25.7	<.0001	****	.180	117	24.8	<.0001	****	.175	117	26.0	<.0001	****	.182
C	1	117	154.9	<.0001	****	.570	117	162.0	<.0001	****	.580	117	173.1	<.0001	****	.597
T×C	1						117	8.98	.003	**	.071	117	8.70	.003	**	.069
Session 2																
T	1	111	29.0	<.0001	****	.207	111	13.4	.0003	***	.108	111	15.1	.0002	***	.120
C	1	111	90.7	<.0001	****	.450	111	156.4	<.0001	****	.586	111	125.9	<.0001	****	.531
T×C	1						111	11.2	.001	**	.093	111	9.58	.002	**	.079

T = task (ignore, suppress); C = condition (control, lure); S = session (1, 2). *F* ratios with *p* ≥ .10 are omitted. *R*^2^_β_ represents the semi-partial *R*^2^ estimate of effect size. (*)*p* ≤ .1, ^*^p ≤ .05, ^**^p ≤ .01, ^***^p ≤ .001, ^****^p ≤ .0001.

#### Sensitivity

Highly significant main effects of task and condition were found across sessions as well as for each session, stemming from better performance sensitivity for the ignore than for the suppress task, and for control than for lure trials (Fig. 2A; see Supplementary Fig. S2 for box plots with subject-level data). The across-session analysis also revealed a significant session main effect due to better performance in Session 2, and a marginal Condition × Session interaction that resulted from greater session improvements of lure (*M* ± *SD*, Session 1 vs. 2: 3.38 ± 0.26 vs. 4.09 ± 0.26; simple effect of session, *p* = .0001) than control trials (5.47 ± 0.26 vs. 5.73 ± 0.26; n.s.).

#### Median response latency

Highly significant effects of task, condition, and Task × Condition were found across sessions and for each session (Table 3), with main effects originating from faster correct responses for ignore than suppress, and for control than lure (Fig. 2B; see Supplementary Fig. S3 for corresponding box plots). The Task × Condition interactions stemmed from greater task differences for lure than control trials (simple effects of task, across sessions and for each session, at lure, all *p* < .0001; at ignore, all n.s.; gray difference bars in Fig. 2B). Furthermore, a session main effect was due to overall faster responses in Session 2 than in Session 1, but there were no interactions of session with task or condition.

#### Inverse efficiency

All effects revealed by the mixed-model analysis closely paralleled those observed for response latencies (Table 3, Fig. 2C; see Supplementary Fig. S4).

### Suppress versus remember

Table 4 provides a summary of mixed-model effects comparing suppress and remember tasks, analogous to Table 3.

**Table 4:** Summary of linear mixed-effect regression models comparing suppress and remember tasks

		Sensitivity (*d*_L_)					Median Response Latency					Inverse Efficiency				
	*df*_1_	*df*_2_	*F*	*p*		*R*^2^_β_	*df*_2_	*F*	*p*		*R*^2^_β_	*df*_2_	*F*	*p*		*R*^2^_β_
Sessions 1 & 2																
T	1	265.2	5.63	.018	*	.021	264.9	31.4	<.0001	****	.106	265	8.35	.004	**	.031
C	1	265.2	276.5	<.0001	****	.510	264.9	663.8	<.0001	****	.715	265	608.7	<.0001	****	.697
S	1	266.6	13.7	.0003	***	.049	267.0	10.9	.001	**	.039	267	10.8	.001	**	.039
T×C	1						264.9	8.6	.004	**	.031					
T×S																
C×S																
T×C×S																
Session 1																
T	1	117	2.88	.092	(*)	.024	117	10.9	.001	**	.085					
C	1	117	184.5	<.0001	****	.612	117	320.3	<.0001	****	.732	117	325.2	<.0001	****	.735
T×C	1						117	3.77	.054	(*)	.031					
Session 2																
T	1	111	3.66	.058	(*)	.032	111	18.9	<.0001	****	.146	111	5.52	.021	*	.047
C	1	111	138.2	<.0001	****	.555	111	300.2	<.0001	****	.730	111	245.1	<.0001	****	.688
T×C	1						111	4.27	.041	*	.037					

T = task (suppress, remember); C = condition (control, lure); S = session (1, 2). *F* ratios with *p* ≥ .10 are omitted. *R*^2^_β_ represents the semi-partial *R*^2^ estimate of effect size. (*)*p* ≤ .1, ^*^p ≤ .05, ^**^p ≤ .01, ^***^p ≤ .001, ^****^p ≤ .0001.

#### Sensitivity

The across session model revealed significant main effects of both task and condition. These effects were driven by better performance sensitivity for the remember than for the suppress condition, as well as better performance for control than for lure trials (Fig. 2A, Fig. S2). A main effect of session was also found in the across-session model, stemming from better performance sensitivity at Session 2. Analyses run separately for each session revealed a significant main effect of condition—however, the main effect of task seen in the across-session model dropped to marginal significance.

#### Median response latency

Highly significant effects of task, condition, and Task × Condition were found in the across-session model (Table 4). The main effects were driven by faster correct responses for suppress than for remember, and faster correct responses for control than for lure (Fig. 2B, Fig. S2). The Task × Condition interaction was driven by larger task differences for lure than for control trials (simple main effect of task, at lure, *p* < .0001; at remember, *p* = .06; white difference bars in Fig. 2B). That is, the difference in task performance (suppress vs. remember) was significantly larger for the lure than the control condition. The highly significant main effects of task and condition remained significant in separate analyses for each session, whereas the Task × Condition effect was weaker, being just below (Session 1) or above (Session 2) a conventional level of significance. However, the source of the interaction remained the same (i.e., larger task differences for lure vs. control trials; simple effects of task, Session 1, *p* = .0003 vs. n.s.; Session 2, *p* < .0001 vs. n.s.).

#### Inverse efficiency

The across session model revealed significant main effects of task and condition (Table 4), due to lower inverse efficiency scores for the suppress than for the remember task, as well as for the control than for the lure condition (Fig. 2C, Fig. S3). As for the median response latency, a Task × Condition interaction was driven by larger task differences for lure than for control trials (simple main effect of task, at lure, *p* = .002; at remember, n.s.; see the white difference bars in Fig. 2C). Although the condition main effect remained significant in each session, the task main effect was only significant for Session 2, and no significant Task × Condition interaction emerged in either session.

### Reliability analysis

As would be expected from the almost identical patterns of findings reported above for Sessions 1 and 2, formal test–retest reliability estimates were good or excellent.

With all three tasks included, the Spearman–Brown-corrected correlation coefficients ranged between .89 and .97 for the three behavioral measures (Table 5). Similarly high estimates were observed for the ignore/suppress and suppress/remember task combinations tested here, ranging between .88 and .96. Finally, estimates were also obtained separately for each task, with the rounded Spearman–Brown coefficients ranging between .80 and .95. These findings suggest strong temporal stability of the behavioral paradigmatic effects over a 1-week interval.

**Table 5:** Test–retest reliability (temporal stability across sessions; Spearman–Brown-corrected correlation coefficients of Pearson’s *r*) and internal consistency (at each session; Cronbach’s *α*) of behavioral measures including all tasks, ignore/suppress, and suppress/remember comparisons, and separately for each task

	Sensitivity (*d*_L_)		Median Response Latency		Inverse Efficiency	
Spearman–Brown Coefficient ^a^						
Ignore/suppress/remember	.894 (.809)		.966 (.934)		.968 (.939)	
Ignore/suppress	.877 (.780)		.960 (.923)		.956 (.917)	
Suppress/remember	.891 (.803)		.947 (.899)		.958 (.919)	
Ignore	.796 (.661)		.887 (.797)		.888 (.799)	
Suppress	.868 (.767)		.944 (.894)		.951 (.907)	
Remember	.829 (.707)		.917 (.846)		.940 (.886)	
	Session		Session		Session	
Cronbach’s *α*	1	2	1	2	1	2
Ignore/suppress/remember	.918	.920	.860	.870	.862	.882
Ignore/suppress	.900	.888	.812	.839	.807	.831
Suppress/remember	.896	.915	.802	.801	.803	.831
Ignore	.864	.900	.658	.682	.571	.661
Suppress	.900	.889	.612	.642	.641	.614
Remember	.834	.849	.504	.594	.460	.610

^a^All reliabilities were computed using the Spearman–Brown prophecy formula, *r*_SB_ = *k* × *r*/(1 + (*k* – 1) × *r*), where *r* is the test–retest correlation and *k* the correction factor, which is 2 in all cases (i.e., same number of items in Sessions 1 and 2). Correlations between sessions (Pearson’s *r*) are reported in parentheses.

Internal consistency was measured for each session using Cronbach’s *α* (Table 5). Analyses run on all three tasks yielded Cronbach’s *α* values ranging between .86 and .92, and analyses of the ignore/suppress and suppress/remember combinations revealed values ranging from .80 to .92. In contrast, separate estimates of Cronbach’s *α* for each task were dependent on the performance measure. Whereas good or excellent internal consistencies were observed for each task for the sensitivity measure *d*_L_ (.83 ≤ *α* ≤ .90), values for the two behavioral indices that involved response latency (RT_med_) ranged between unacceptable and questionable (.46 ≤ *α* ≤ .68). Overall, we found good to excellent internal consistencies of the behavioral measures if paradigm-related effects were considered (i.e., including at least two tasks).

## Discussion

To investigate the role of proactive control during auditory processing, which would be of particular value in studies of cognitive function in schizophrenia (e.g., Barch & Ceaser, 2012), this study introduced an auditory analogue of visual ignore/suppress WM tasks (Nee & Jonides, 2008, 2009; Smith et al., 2011). Previous work has suggested that proactive control may be a common mechanism driving multiple cognitive deficits in schizophrenia (Barch & Ceaser, 2012). However, there is ample evidence that patients with schizophrenia, in particular those experiencing severe and frequent hallucinations, are more impaired in the auditory than the visual modality (e.g., Ford, 1999; Kayser et al., 2009; Pelletier et al., 2005), and yet most cognitive research has focused and continues to rely on visual tasks. The specific goal of the present report was to evaluate in a sample of healthy adults whether (1) the behavioral performance observed for these new auditory WM tasks would match those typically seen in the visual domain, and (2) to determine the reliability of these behavioral effects (i.e., their internal consistency and temporal stability over a 1-week time interval). We also added a third item-recognition-like remember task to the two ignore/suppress tasks to provide a direct control condition for the suppress task, which may have higher cognitive demands than the ignore task (i.e., WM load, reliance on source memory). Hence, this report evaluated whether these new auditory WM tasks would be suitable for the study of cognitive control in schizophrenia.

The present findings strongly suggest that these new auditory WM tasks are ideally suited for these purposes. Aside from the expected superior performance for ignore versus suppress tasks, and poorer performance for lure versus control trials, we found that response latencies were particularly slower for lure than control trials during the suppress than during the ignore task. Importantly, the predicted Task × Condition interaction was present in both test sessions. Although this effect was not significant for performance accuracy, the means were nonetheless in the same direction, thereby yielding significant Task × Condition interactions for the combined inverse efficiency measure for each session. The pattern of findings is highly comparable to that observed during visual ignore/suppress tasks (Nee & Jonides, 2008, 2009; Smith et al., 2011), strongly suggesting that proactive control processes can be effectively examined in the auditory domain with healthy adults. The response latencies reported here (i.e., ranging between 885 and 1,646 ms) were longer than those reported previously for visual ignore/suppress tasks (e.g., mean reactions times ranged between 708 and 898 ms for healthy control participants in Smith et al., 2011). This is likely due to the use of auditory as opposed to visual stimuli, which require at least the full stimulus duration (about 400 ms) as additional processing time, and also to greater cognitive demands. The latter point is underscored by prolonged lure-minus-control differences in response latency for the auditory tasks (about 300 ms here vs. less than 200 ms reported by Smith et al., 2011) and substantially higher false alarm (error) rates (about 3–4 times than those of Smith et al., 2011). Indeed, condition effects were also strong for the ignore task, which in the visual modality can be small and sometimes fragile (e.g., Nee & Jonides, 2008; Smith et al., 2011)—this increased robustness emphasizes the value of both auditory tasks for patient populations.^8^ Nonetheless, the behavioral effects were highly reproducible across test sessions separated by 1 week. Although repeated testing revealed strong evidence of practice effects, yielding more accurate and faster responses in Session 2, session effectively failed to interact with task and condition, thereby supporting the assertion of robust, stable and replicable paradigmatic effects.

With regard to our secondary objective, that is, including a remember task as a direct control for the suppress task, we likewise observed highly similar effects in both test sessions, with significant session main effects merely indicative of overall better performance due to practice. However, we found moderate evidence to support the idea that the suppress task has a behavioral advantage over the remember task. Although both tasks require encoding of all four items and relevant contextual (source) information, *only* the suppress task explicitly requires, or rather allows for, inhibition of task-irrelevant items during the maintenance interval. This was reflected in significantly shorter response latencies than for the remember task, which would be expected given that only two items, as opposed to four, need to be compared to the probe in the suppress task. Moreover, this task advantage was most pronounced for lure than control trials. On the other hand, a marginally better performance sensitivity was seen for remember versus suppress, which may be interpreted in light of the task differences in the probe. Whereas the suppress task involved a binaural probe (as did the ignore task), which requires a certain conceptual transformation when comparing the probe to the monaural encoding items, no such transformation was needed for the remember task for which the probe was, in case of a match, physically identical to the encoding item (i.e., identity of content–context binding). This stimulus identity may have facilitated item comparisons through an increase in discriminability between content–context bindings in WM (Cyr et al., 2017) and likely contributed to a reduced false alarm rate. Although the interpretation of these behavioral results is thereby complicated by the differences in probe between suppress and remember tasks—binaural versus monaural presentations—the trial sequence up to the probe onset was virtually identical in the suppress and remember tasks; similar behavioral performance in suppress and remember tasks may therefore be helpful for the interpretation of concurrent electrophysiological measures.

Finally, as might be expected from the preservation of findings across test sessions, formal estimates of both internal consistency and test–retest reliability were good to excellent, indicating that the behavioral measures obtained during these new auditory WM tasks were both robust (i.e., internally consistent) and stable across a 1-week testing interval. Remarkably, reliabilities approached or even exceeded the desirable standard of .95 recommended for applied settings (Nunnally, 1978), possibly affording clinical utility of these behavioral measures. The reliability of these effects was not compromised by notable practice effects (i.e., participants’ task performance improved at retest). These results add further support to the conclusion that proactive control can be effectively measured with the auditory ignore/suppress/remember paradigm. However, the poor Cronbach’s *α* values observed for response latency when tasks were assessed in isolation (i.e., excluding task-related effects) should caution future studies (or applications) from relying exclusively on latency measures when employing only one of these tasks at a time.

### Limitations

We note that the critical Task × Condition interactions for the ignore/suppress and the suppress/remember comparisons are noncrossover and therefore ambiguous with respect to the interpretation of the latent psychological process (Loftus, 1978). Noncrossover interactions are tied to the scale of measurement of the dependent variable, which allows for the possibility of applying a nonlinear transformation of the monotonic increase to remove the interaction. Although this interpretational issue is largely unrecognized in the literature and among experimental psychologists (Wagenmakers, Krypotos, Criss, & Iverson, 2012), the robustness of the conclusions may be bolstered by using several plausible transformations of the dependent measure. While the present study included accuracy, response latency, and their combination as inverse efficiency as separate estimates of behavioral performance, with all—although to different degrees—warranting the same conclusions, we further addressed the interpretational ambiguity by employing other common estimates of response latency (i.e., mean, log_10_-transformed, and trimmed means). The corresponding analyses of these response latency estimates were virtually identical to the reported findings for median RT (see Supplementary Fig. S5), thereby convincingly mitigating this concern.

### Future directions and considerations

A major advantage of this auditory WM paradigm is that electrophysiological and other neurobiological measures can be recorded during encoding, maintenance, and retrieval of information in WM, as these processing stages are distinctly separated in time. The sequential nature of these tasks, with their virtually identical structure during the encoding, maintenance, and retrieval phases, is therefore ideal for evaluating the extent to which behavioral deficits are related to disturbance in early sensory/perceptual processing, maintenance of information, or memory retrieval (e.g., Kayser et al., 2006; Kayser et al., 2017). Concurrent electrophysiological measures (ERPs, EROs) can provide even more meaningful insights from these three auditory WM tasks by examining the ongoing brain activity during encoding and maintenance intervals (e.g., Kayser et al., 2017; Kayser, Tenke, Wong, Alvarenga, et al., 2018a; Kayser, Tenke, Wong, Casal-Roscum, et al., 2018b). The use of these methods will allow the examination of brain activity at each stage of processing, with each stage reflecting a sound manipulation of sensory/perceptual and attentional control processes during WM, as opposed to behavioral results that reflect a summation of cognitive activity during the entire task sequence. Systematic study employing targeted outcome measures should help gain a deeper understanding of the brain mechanism underlying rapidly unfolding cognitive control processes in WM. Of course, the specific arrangement of the different processing stages is not restricted to auditory stimuli but may likewise be used with visual stimuli (e.g., Nee & Jonides, 2008). Employing this paradigm with different stimulus categories, such as words, objects, or environmental sounds, or systematic alteration of WM load or retrieval difficulty, could provide meaningful extensions, particularly when used in combination with psychophysiological and/or neuroimaging measures.

## Conclusions

The present study introduced a new auditory WM paradigm that closely emulates visual ignore/suppress WM tasks. The findings, which were remarkably stable over test–retest sessions separated by one week, are consistent with those of prior reports using visual tasks, showing poorer behavioral performance for the rejection of lure than control items, and this condition-related effect was greater in the suppress than ignore task. Findings for a third item-recognition-like (remember) task suggested that these task-by-condition effects are not merely caused by a higher WM load or greater reliance on contextual (source) memory in the suppress task, providing indirect behavioral support for actively inhibiting or discarding encoded items from WM and thus for the existence of an active forgetting process (e.g., Anderson et al., 2004). Accordingly, the auditory ignore/suppress/remember paradigm may be a useful tool for examining the role of proactive control within the context of WM (Kayser, Tenke, Wong, Casal-Roscum, et al., 2018b), and may be particularly helpful for investigating cognitive control processes in schizophrenia. In fact, preliminary findings by our group suggest that employing these tasks with schizophrenic patients is both feasible and promising (Kayser, Tenke, Wong, Alvarenga, et al., 2018a). Future work should focus on using this auditory WM paradigm in conjunction with electrophysiological measures and psychiatric populations, specifically those diagnosed with psychosis.

## Electronic supplementary material

ESM 1 (DOCX 1.54 MB)

## Footnotes

1 These electrophysiological findings will be reported elsewhere.

2 The sample size calculation was determined a priori given preliminary data available from four healthy pilot participants, revealing large effect sizes that would provide sufficient power (> 90%) with *N* = 40 (NIMH award MH106905).

3 Because the present paradigm was designed for simultaneous EEG recordings, it was mandatory to maintain the same event sequence for all task conditions, including the two cues that preceded and followed the letter encoding series. Accordingly, the second cue does not provide new information during the ignore task, the first cue does not provide meaningful information during the suppress task (other signaling the start of the trial), and both cues provide no task-relevant information during the remember task. Although it may be argued that the second cue during ignore may act as a “suppress” cue, given that no new information is provided at that time, this seems rather unlikely. Although it is difficult to contemplate this issue solely on the basis of the behavioral data, the electrophysiological findings strongly suggest a valid operationalization of bottom-up cognitive control processes during the ignore task (Kayser et al., 2017; Kayser, Tenke, Wong, Alvarenga, et al., 2018a; Kayser, Tenke, Wong, Casal-Roscum, et al., 2018b).

4 All stimuli, permutation sequences, and training trials, along with their Presentation scripts and setup files, are available for download (Supplement A).

5 Because the sensitivity measure *d*_L_ can vary between +8.3 (perfect performance), 0.0 (chance), and –8.3 (systematic failure), an arbitrary constant of 10 was added to the denominator to avoid problems with sign inversion, value inflation or division by zero.

6 Additional mixed models also included age and gender as fixed factors. All effects reported below were preserved in these analyses. For sake of brevity, these confirmatory findings will not be detailed further.

7 The auditory WM paradigm, as performed at one test session, was conceived as a “test,” which was repeated after 1 week.

8 To compare effect sizes across modalities, we used the means and standard deviations for visual ignore/suppress tasks, as reported for healthy adults in Smith et al. (2011) for response latencies and error rates, to compute Cohen’s *d* as a measure of effect size (Cohen, 1988). Analogous effect sizes were then computed for auditory ignore/suppress tasks in Sessions 1 and 2 (see Table 3). The effect sizes, as calculated for the condition effect (lure vs. control) during each task (ignore, suppress), were notably smaller for the visual (0.12 ≤ *d* ≤ 0.75) than for the auditory tasks (0.93 ≤ *d* ≤ 1.91).
