# Supplementary material for: Behavioral measures of attention and cognitive control during a new auditory working memory paradigm
Source: Behav Res Methods. 2019 Dec 3;52(3):1161–74. doi: 10.3758/s13428-019-01308-z (PMC7266708; doi:10.3758/s13428-019-01308-z)
Supplement: Supplementary file 1 — (DOCX 1.54 MB) [file 13428_2019_1308_MOESM1_ESM.docx]

**Supplementary Material**

| Table T1. Interclass correlations (rho [*ρ*]) of mixed-model baseline analyses for each behavioral measure, planned task comparisons, and test session. | | | | | | | | | | | | | | | |
| --- | --- | --- | --- | --- | --- | --- | --- | --- | --- | --- | --- | --- | --- | --- | --- |
|  |  |  | | Sensitivity (*d*_L_) | | | |  | Median response latency | | |  | Inverse efficiency | | |
|  | *N* | |  | | *ρ* | *t* | *p* |  | *ρ* | *t* | *p* |  | *ρ* | *t* | *p* |
| Sessions 1 & 2 |  | |  | |  |  |  |  |  |  |  |  |  |  |  |
| Ignore/Suppress | 312 | |  | | .417 | 8.08 | <.0001 |  | .319 | 5.92 | <.0001 |  | .309 | 5.73 | <.0001 |
| Suppress/Remember | 312 | |  | | .456 | 9.01 | <.0001 |  | .194 | 3.49 | .0003 |  | .236 | 4.28 | <.0001 |
| Session 1 |  | |  | |  |  |  |  |  |  |  |  |  |  |  |
| Ignore/Suppress | 160 | |  | | .378 | 5.14 | <.0001 |  | .212 | 2.72 | .004 |  | .178 | 2.28 | .012 |
| Suppress/Remember | 160 | |  | | .397 | 5.44 | <.0001 |  | .097 | 1.22 | .111 |  | .095 | 1.20 | .115 |
| Session 2 |  | |  | |  |  |  |  |  |  |  |  |  |  |  |
| Ignore/Suppress | 152 | |  | | .459 | 6.33 | <.0001 |  | .263 | 3.33 | .0005 |  | .282 | 3.60 | .0002 |
| Suppress/Remember | 152 | |  | | .519 | 7.43 | <.0001 |  | .074 | 0.91 | .183 |  | .176 | 2.19 | .015 |

*Note*. *N*: number of observations. *t* statistics (with one-tailed *p* values) report ICC (*ρ*) differences from zero. The generic R code to compute unconditional ICCs for a baseline model (i.e., with no explanatory variables in the equation; Bickel, 2007; McGraw & Wong, 1996) and to convert *ρ* to its corresponding *t* and *p* values, along with the data, is provided as Supplement B.

| Table T2. Summary of linear mixed effect regression models comparing Ignore, Suppress, and Remember tasks across test sessions. | | | | | | | | | | | | | | | | |
| --- | --- | --- | --- | --- | --- | --- | --- | --- | --- | --- | --- | --- | --- | --- | --- | --- |
|  |  | Sensitivity (*d*_L_) | | | | | Median response latency | | | | | Inverse efficiency | | | | |
|  | *df*_1_ | *df*_2_ | *F* | *p* |  | *R*^2^ | *df*_2_ | *F* | *p* |  | *R*^2^ | *df*_2_ | *F* | *p* |  | *R*^2^ |
| T | 2 | 417.1 | 25.1 | < .0001 | *** | .107 | 415.7 | 69.8 | < .0001 | *** | .251 | 417.1 | 48.1 | < .0001 | *** | .187 |
| C | 1 | 417.1 | 327.1 | < .0001 | *** | .440 | 415.7 | 702.6 | < .0001 | *** | .628 | 417.1 | 648.1 | < .0001 | *** | .608 |
| S | 1 | 419.1 | 21.4 | < .0001 | *** | .049 | 418.1 | 13.2 | .0003 | *** | .031 | 419.4 | 14.1 | .0002 | *** | .033 |
| T*C | 2 |  |  |  |  |  | 415.7 | 28.4 | < .0001 | *** | .120 | 417.1 | 20.2 | < .0001 | *** | .088 |
| T*S |  |  |  |  |  |  |  |  |  |  |  |  |  |  |  |  |
| C*S |  |  |  |  |  |  |  |  |  |  |  |  |  |  |  |  |
| T*C*S |  |  |  |  |  |  |  |  |  |  |  |  |  |  |  |  |

Note. T = Task (Ignore, Suppress, Remember); C = Condition (Control, Lure); S = Session (1, 2). *F* ratios with *p* ≥ .10 are omitted.

*R*^2^ represents the semi-partial *R*^2^ estimate of effect size. (*) *p* ≤ .1, * *p* ≤ .05, ** *p* ≤ .01, *** *p* ≤ .001, **** *p* ≤ .0001.

| **Table T3.** Means (±SD) and effect sizes (Cohen’s *d*) of behavioral performance measures for condition effects (Lure/Control) during visual and auditory implementations of Ignore/Suppress tasks. | | | | | | | | |
| --- | --- | --- | --- | --- | --- | --- | --- | --- |
|  |  |  | **Ignore** | |  | **Suppress** | |  |
|  |  |  | **Lure** | **Control** | ***d*** | **Lure** | **Control** | ***d*** |
| **Visual** | ***N* = 24** ^a^ | **RT_mean_** | 741  ±189 | 708  ±174 | .181 | 898  ±311 | 720  ±195 | .685 |
|  |  | **ER** | 1.9  ±4.0 | 0.7  ±2.0 | .379 | 6.1  ±7.0 | 1.8  ±4.0 | .754 |
| **Auditory** | ***N* = 40** ^b^ | **RT_med_** | 1320  ±483 | 923  ±180 | 1.08 | 1646  ±474 | 1004  ±182 | 1.78 |
|  |  | **ER** | 19.1  ±17.0 | 1.7  ±3.7 | 1.41 | 27.9  ±17.2 | 3.1  ±6.3 | 1.91 |
|  |  | ***d*_L_** | 3.9  ±2.0 | 5.8  ±1.3 | .830 | 2.9  ±2.2 | 5.1  ±1.6 | 1.14 |
|  | ***N* = 38** ^c^ | **RT_med_** | 1243  ±499 | 885  ±214 | .932 | 1518  ±494 | 897  ±183 | 1.66 |
|  |  | **ER** | 13.3  ±14.0 | 1.7  ±3.1 | 1.14 | 21.2  ±18.2 | 2.4  ±3.9 | 1.42 |
|  |  | ***d*_L_** | 4.6  ±2.1 | 6.0  ±1.5 | .767 | 3.5  ±2.1 | 5.3  ±1.6 | .964 |
| *Note*. RT_mean_ = mean response latency; RT_med_ = median response latency; ER = error rate; *d*_L_ = sensitivity (Snodgrass & Corwin, 1988); *d* = effect size (Cohen, 1988). ^a^ Data from Smith et al. (2011), Table 2; ^b^ Data from session 1; ^c^ Data from session 2. | | | | | | | | |

*Supplementary Figure Captions*

**Figure S1.** Amplitude waveforms of 15 letter sounds (A to F, I to O, R to U) and one cue (‘buzz’) stored in digital WAV file format (48,000 samples/s, 550 ms duration). An individual stimulus duration was determined by identifying the time points when the signal amplitude first and last exceeded an arbitrary threshold (7.5 × 10-5), as indicated by horizontal brackets. Note that within each sound file, signal amplitudes are preceded by approximately 50 ms of silence.

**Figure S2**. Box and whisker plots of response sensitivity [*d*_L_] for each condition (Control, Lure) and task (Ignore, Suppress, Remember), separately plotted for each session. Plots were constructed using the geom-boxplot function in the ggplot2 package within R (Wickham, 2016). Dots represent individual participant data points (i.e., per-subject/condition means). Boxes represent first (Q1) and third (Q3) quartiles of the overall dataset. Horizontal black lines through the body of the box represent the median. Upper whiskers extend to Q3 + 1.5 * inter-quartile range (IQR), while lower whiskers extend to Q1 – 1.5*IQR.

**Figure S3**. Box and whisker plots of median response latency [ms] as in Fig. S1.

**Figure S4**. Box and whisker plots of inverse efficiency [ms / (*d*_L_ + 10)] as in Fig. S1.

**Figure S5.** Mean (±SEM) response latency [ms] for four different data transformations: mean, median, mean log_10_ (back-transformed to ms), and trimmed mean (after discarding 10% at each end of the individual distribution). Data were pooled across session and are shown for each condition (Control, Lure) and task (Ignore, Suppress, Remember). Condition means (line graphs) are supplemented by mean difference scores (bar graphs) showing Ignore-minus-Suppress and Suppress-minus-Remember. Graphs depict raw data.


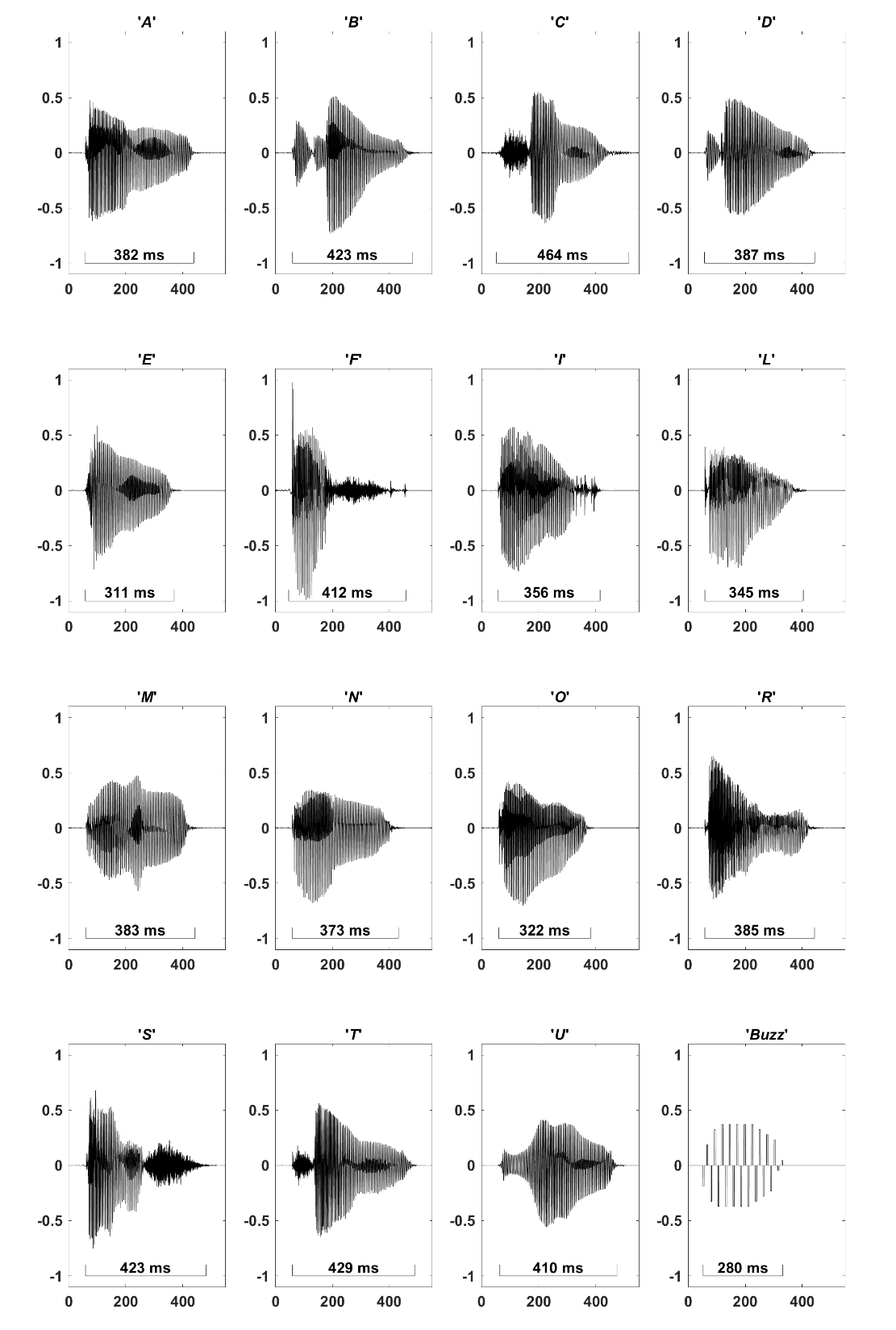


**Figure S1.** Amplitude waveforms of 15 letter sounds (A to F, I to O, R to U) and one cue (‘buzz’) stored in digital WAV file format (48,000 samples/s, 550 ms duration). An individual stimulus duration was determined by identifying the time points when the signal amplitude first and last exceeded an arbitrary threshold (7.5 × 10^-5^), as indicated by horizontal brackets. Note that within each sound file, signal amplitudes are preceded by approximately 50 ms of silence.


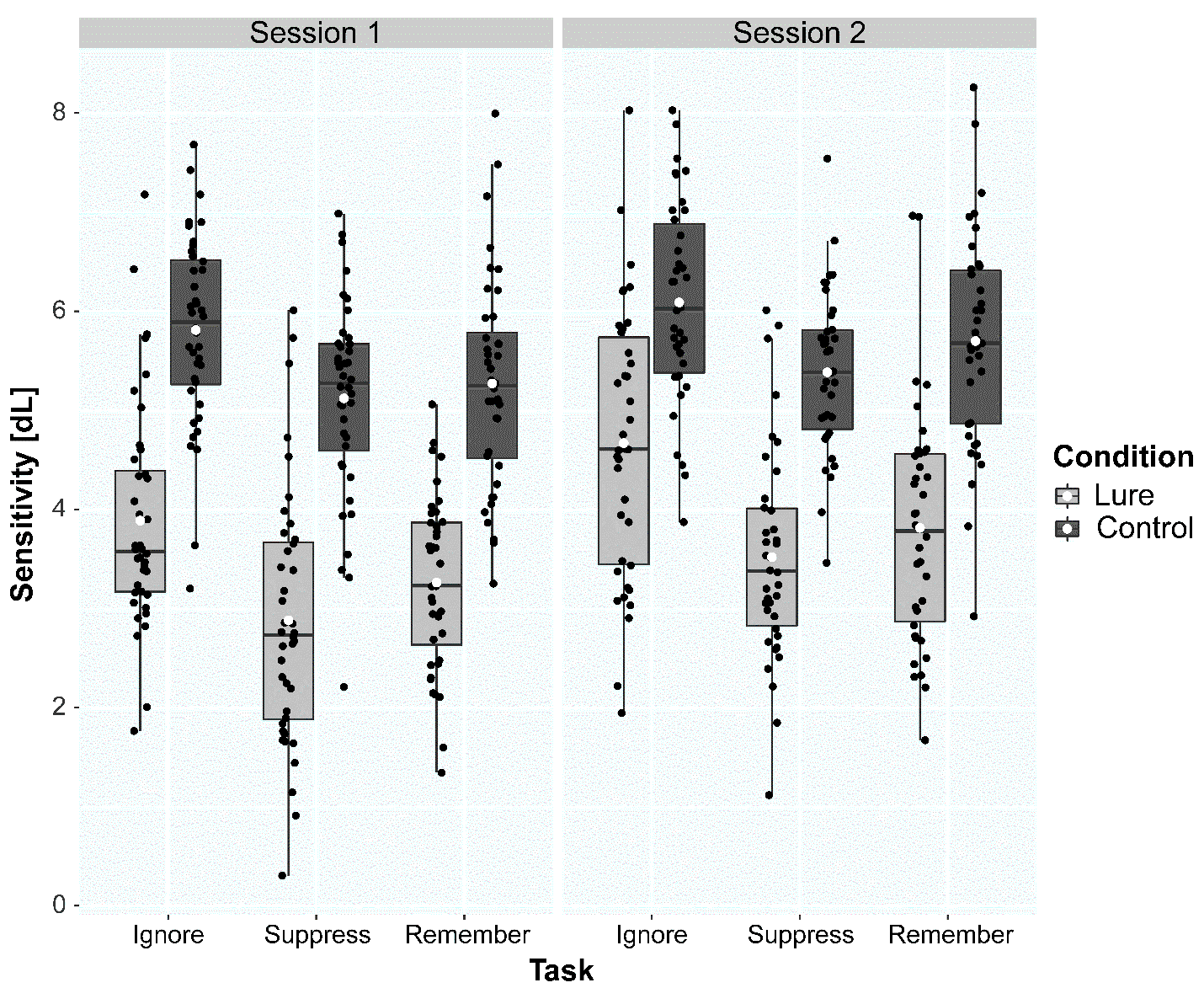


**Figure S2.** Box and whisker plots of response sensitivity [d_L_] for each condition (Control, Lure) and task (Ignore, Suppress, Remember), separately plotted for each session. Plots were constructed using the geom-boxplot function in the ggplot2 package within R (Wickham, 2016). Dots represent individual participant data points (i.e., per-subject/condition means). White dots represent group means. Boxes represent first (Q1) and third (Q3) quartiles of the overall dataset. Horizontal black lines through the body of the box represent the median. Upper whiskers extend to Q3 + 1.5 * inter-quartile range (IQR), while lower whiskers extend to Q1 – 1.5*IQR.


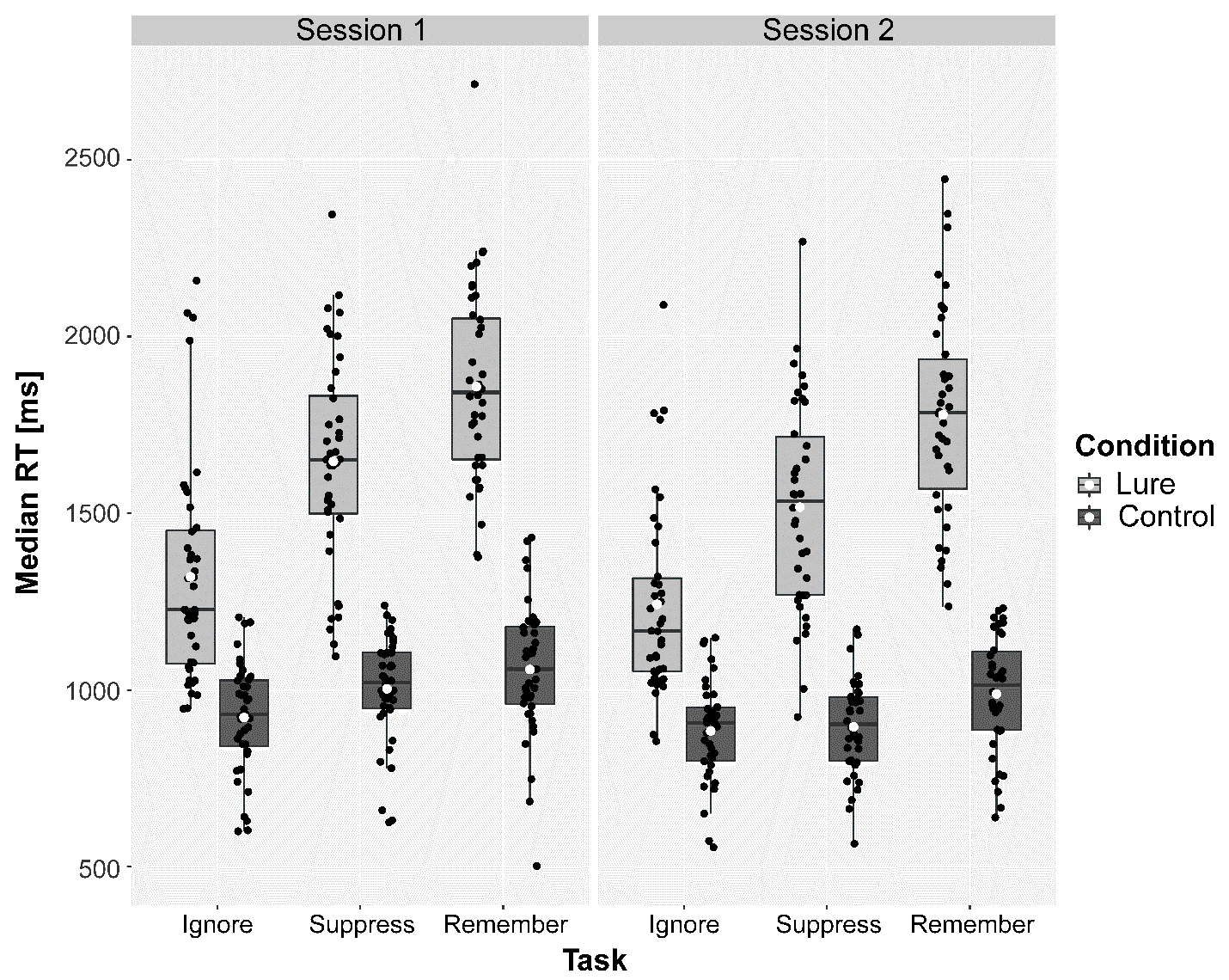


**Figure S3.** Box and whisker plots of median response latency [ms] as in Fig. S2.


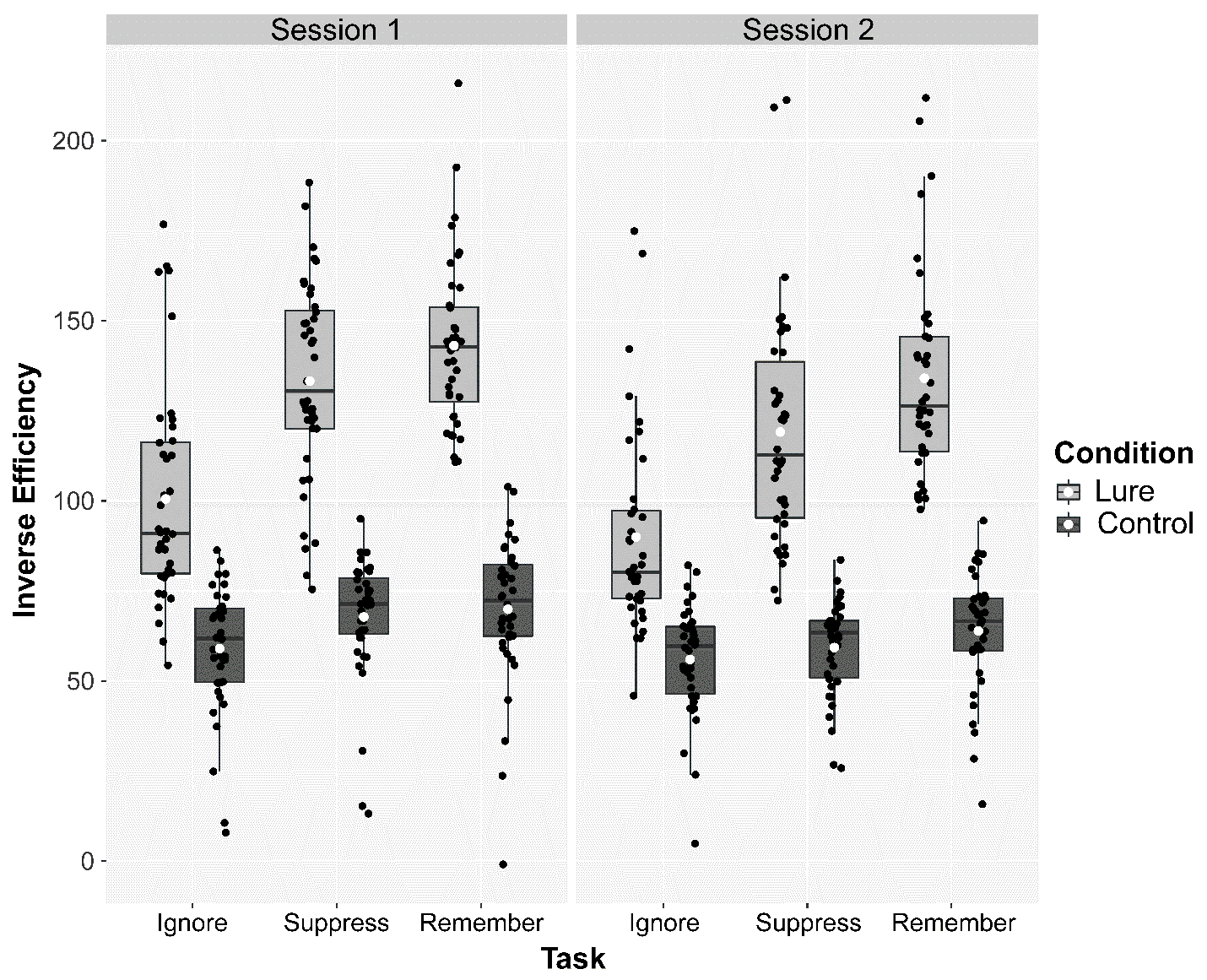


**Figure S4.** Box and whisker plots of inverse efficiency [ms / (*d*_L_ + 10)] as in Fig. S2.


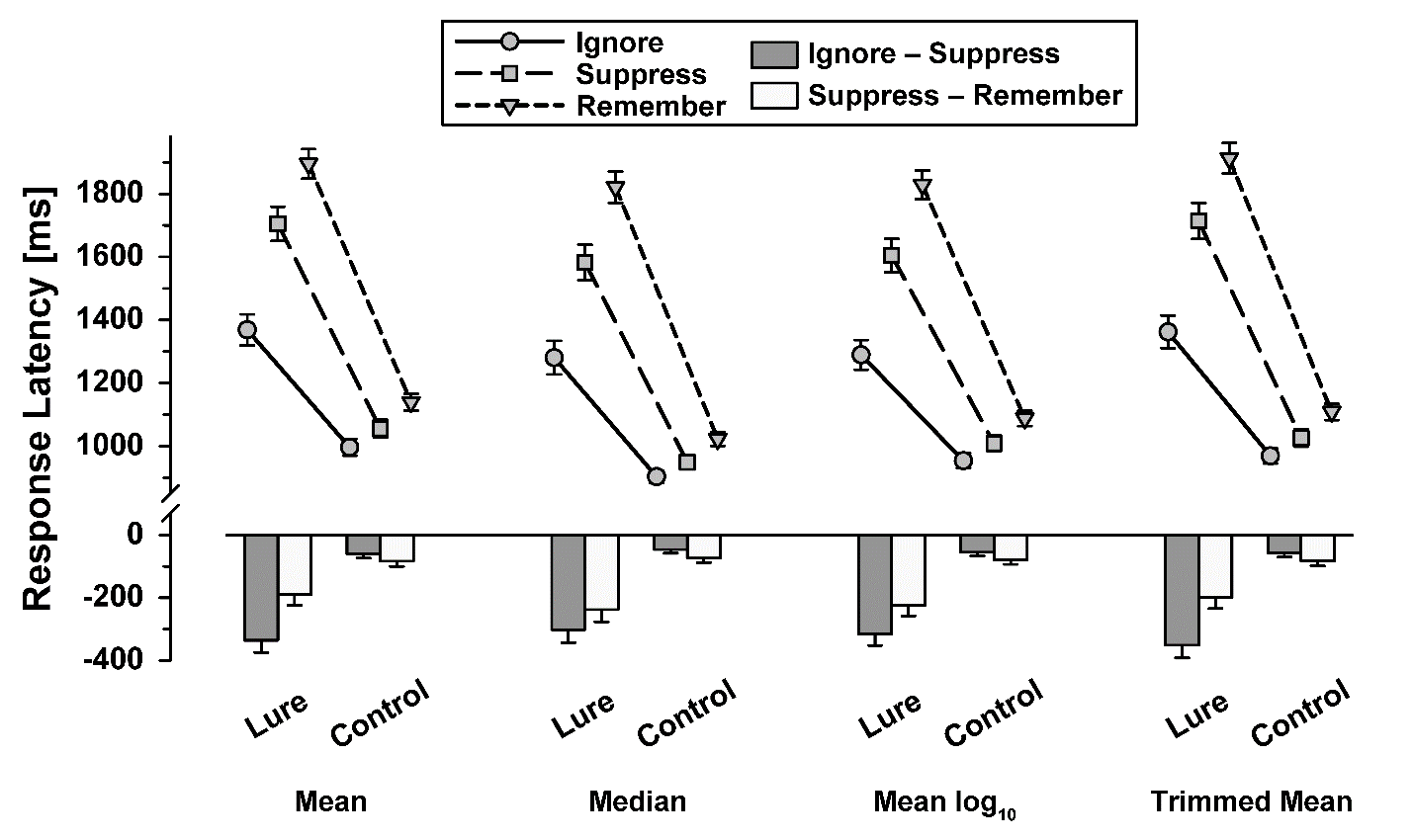


**Figure S5.** Mean (±SEM) response latency [ms] for four different data transformations: mean, median, mean log_10_ (back-transformed to ms), and trimmed mean (after discarding 10% at each end of the individual distribution). Data were pooled across session and are shown for each condition (Control, Lure) and task (Ignore, Suppress, Remember). Condition means (line graphs) are supplemented by mean difference scores (bar graphs) showing Ignore-minus-Suppress and Suppress-minus-Remember. Graphs depict raw data.

**Supplement A**

All sounds (.wav file format), graphic stimuli (.pcx file format), and Presentations® stimulation sequences and scripts (.sce, .pcl, .tem and .exp files) are available for download (archive *BRM_Kayser_etal_2019_Procedure.zip*; see text file *Readme.txt* included in archive for further documentation).

**Supplement B**

The R code syntax used for all mixed effect models and a spread sheet containing the behavioral data are available for download (archive *BRM_Kayser_etal_2019_R.zip*; see comments in R script for further documentation).

REFERENCE

Wickham, H. (2016). *ggplot2: Elegant graphics for data analysis*. New York, NY: Springer.
